# Electrophoretic Pattern and Antibacterial Activity of Proteins from Vicia Faba Seed Extract

**Published:** 2013

**Authors:** Hassan Hoda, Fatemeh Elmi, Maryam Mitra Elmi

**Affiliations:** 1 *Biological Control Department, National Instituteof Plant Protection, Amol, Iran.*; 2 *Department of Chemistry, University of Mazandaran, PO Box: 47416–95447, Babolsar, Iran.*; 3 *Cellular and Molecular Biology Research Center, Babol University of Medical Sciences, Babol, Iran.*

**Keywords:** Antibacterial peptide, antimicrobial peptide, plant, *Vicia faba*

## Abstract

Antibiotic resistance makes Antimierobial peptides (AMPs) agents an alternative for treatment of pathogenic diseases. They are isolated from various invertebrate animals, vertebrates and plants. The present study shows the electrophoretic pattern of protein and peptides from *Vicia faba* seed and reports our first attempt to study the antibacterial activity of *Vicia faba* seed extract. The crude extract electrophoresis was carried out on 12% SDS- PAGE gel. Antibacterial activity on *E. Coli* and *B. Subtilis* from hospital infection was tested and evaluated by measuring the inhibition zone diameter observation. The SDS- PAGE gel electrophoresis shows that the crude extract contains many proteins and peptides with different molecular weight. The inhibition zone was not observed in antibacterial properties tests. Thus, our experiments don’t show any antibacterial activity on *E. Coli* and *B. Subtilis* from hospital isolates. Some other AMPs haven’t also shown any antimicrobial properties on clinical trial. The antibacterial activity of the crude *Vicia faba* seed extract should also be tested on standard bacteria.

Most living organisms are steadily endangered to potentially harmful pathogens through contact, ingestion and inhalation (1). Antibiotics have used to treat pathogenic diseases. During several decades, inappropriate usage of antibiotics and the ability of pathogens to quickly develop resistance mechanisms turn them to less effective agents. Thus, it is necessary to find new biomedical alternatives for pathogenic illness treatment. Antimicrobial peptides (AMPs) are best candidate for this purpose (2-3).

Important substances of the natural defenses against these pathogens. AMPs have been isolated from a wide variety of sources, invertebrates, vertebrates and plants. AMPs are a heterogeneous class of low molecular mass proteins with molecular mass below 25–30 kDa. Most AMPs have hydrophobic and cationic properties (1, 4-5). They exhibit bactericidal, fungicidal, virucidal and tumoricidal properties (6). AMPs have been isolated from a variety of plants (2, 7-14). Fabatin is one of the AMPs peptides that was isolated from *Vicia faba.* Fabatin can affect Gram-negative and Gram- positive bacteria (12). The present study is our attempt to study the electrophoretic pattern of peptides from *Vicia faba* seed extract and its antibacterial activity on *E. Coli* and *B. Subtilis* isolated from hospital samples.

## Materials and Methods

Seeds of* Vicia faba* were purchased from local store. *E. coli* and *B. subtilis* isolated from hospital were gifted by microbiology laboratory, paramedical department, Babol University of medical sciences. Dialysis tubing cellulose membrane with molecular weight cut off 1000 Da. was from Medical Industries, Tehran, Iran. All other chemicals were analytical grade. 

**Extraction of seed proteins**

The extraction of antibacterial protein(s) from seeds was carried out as described previously (14-15). 100 g seeds were grinder in a grinder and then extracted in a buffer (10 mM Na_2_HPO_4_, 15 mM NaH_2_PO_4_, 100 mM KC1, 2 mM EDTA, 1.5% PVPP) by stirring overnight at 4°C. Following a 70% ammonium sulphate precipitation, the sample was centrifuged at 10000 rpm and the supernatant was removed. The pellet was re-suspended in distilled water and then dialysed against distilled water using 1000 Da cut-off dialysis tubing and finally adjusted to (pH=7.3) with 50 mM ammonium acetate. The extract was stored at -20°C for SDS-PAGE electrophoresis.

The extract was passed over Sephadex G75 column (300 x 25 mm) equilibrated in 50 mM ammonium acetate (pH= 7. 3). Proteins were eluted with 50 mM ammonium acetate. Elutions were collected in 5ml and the absorbance was measured by UV-spectrometer (Shimadzu, TCC 240) at 280 nm. The eluted samples were sterilized by 0.22 μm syringe - filter.

**Gel Electrophoresis**

**Fig 1:**
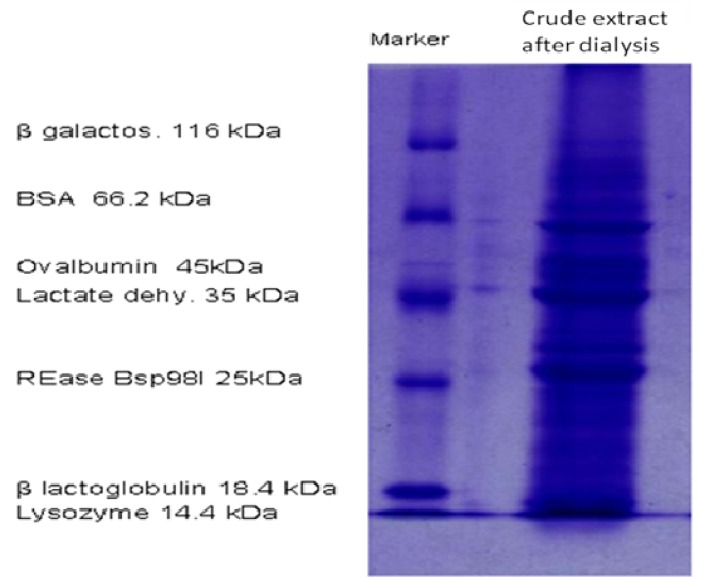
SDS-PAGE electrophoresis of *Vicia faba* seed extract on acryl amide gel %12

After dialysis, SDS-PAGE electrophoresis of the crude extract was carried out on %12 acryl amide gel and stained with Coonmassie blue to check electrophoretic pattern of proteins(Fig. 1) (16).

**Antibacterial Activity of** ***Vicia faba*** **Extract**

**Well Diffusion Agar Method**

One colony of * eaeh E. coli* and *B. subtilis* was separately incubated in 3 ml of LB broth media. Turbidity of the cultured bacteria was compared with 0.5 Mac farland solution. Then 5×10^6 ^CFU were spread on the Muller Hinton agar medium in which holes (diameter of 7 mm) were punched. Then, 100 μl of sterilized crude extract and eluted samples from chromatography were introduced separately to each well. Distillated water was used as a negative control. The plates were incubated at 30°C for 24, 48 and 72 hrs. The antibacterial activity was evaluated by measuring the inhibition zone diameter. The experiment was performed more than three times. 

## Results

The electrophoretic pattern of the crude extract,* Vicia faba*'s seeds, presented different bands. The proteins profiles had different molecular weight ranging from 116 to less than 14 kD (Fig.1). The inhibition zone diameter has not been observed. consequently, growth inhibition of *E. coli* and *B. subtilis* was not observed in the presence of the crude *Vicia faba* seeds extract and the eluted samples from chromatography at any dilutions.

## Discussion

The present study shows that the crude extract of *Vicia faba* seeds contains various proteins and peptides. Also, this study demonstrated that the extract had no antibacterial activity on *E. coli* and *B. subtilis* as a Gram-negative and Gram- positive bacteria respectively. These results are in contrast with a study that purified fabatin from *Vicia faba* seeds and revealed their antibacterial activity (12) and also other studies showing antibacterial activity of several plants (10, 17). It seems that the difference of the source of bacteria could be the main possible explanation. Furthermore, the amount of effective component in our extraction might be very low and therefore more appropriate techniques are necessary to concentrate it. Despite AMPs are a good alternative for treating infections in relation to conventional antibiotics, very few AMPs extracted from plants and animals have been utilized in clinical trials (3, 18) and the bacteria used in the current work, isolated from clinical samples, may be naturally resistant to AMPs (3, 19).

In conclusion this study suffer from some limitations mentioned above and therefore a more appropriate study using standard bacteria and adequate techniques is recommended.
